# Supplementary material for: Microbiomes of three coral species in the Mexican Caribbean and their shifts associated with the Stony Coral Tissue Loss Disease
Source: PLoS One. 2024 Aug 26;19(8):e0304925. doi: 10.1371/journal.pone.0304925 (PMC11346732; doi:10.1371/journal.pone.0304925)
Supplement: S1 File — (ZIP) [file pone.0304925.s001.zip › S3_Table2.docx]

**S3_Table2.** Primer sequences used for amplification of the 16S V3-V4 region.

| **Name of primer** | **Primer sequence** |
| --- | --- |
| Illumina_16S_341F | 5′ -TCGTCGGCAGCGTCAGATGTGTATAAGAGACAG**CCTACGGGNGGCWGCAG** |
| Illumina_16S_805R | 5′-GTCTCGTGGGCTCGGAGATGTGTATAAGAGACAG**GACTACHVGGGTATCTAATCC** |
