# Supplementary material for: Microbiomes of three coral species in the Mexican Caribbean and their shifts associated with the Stony Coral Tissue Loss Disease
Source: PLoS One. 2024 Aug 26;19(8):e0304925. doi: 10.1371/journal.pone.0304925 (PMC11346732; doi:10.1371/journal.pone.0304925)
Supplement: S1 File — (ZIP) [file pone.0304925.s001.zip › S7_Table3.docx]

**S7_Table3.** Post-hoc pairwise PERMANOVA.

|  | *S. siderea* | *O. faveolata* |
| --- | --- | --- |
|  |  |  |
|  |  |  |
| *O. faveolata* | F =2.17 |  |
|  | R^2^=0.18 |  |
|  | p=0.007 |  |
| *M. cavernosa* | F =1.96 | F =2.11 |
|  | R^2^=0.18 | R^2^=0.16 |
|  | p=0.026 | p=0.008 |
