# Supplementary figures and images for: Microbiomes of three coral species in the Mexican Caribbean and their shifts associated with the Stony Coral Tissue Loss Disease
Source: PLoS One. 2024 Aug 26;19(8):e0304925. doi: 10.1371/journal.pone.0304925 (PMC11346732; doi:10.1371/journal.pone.0304925)

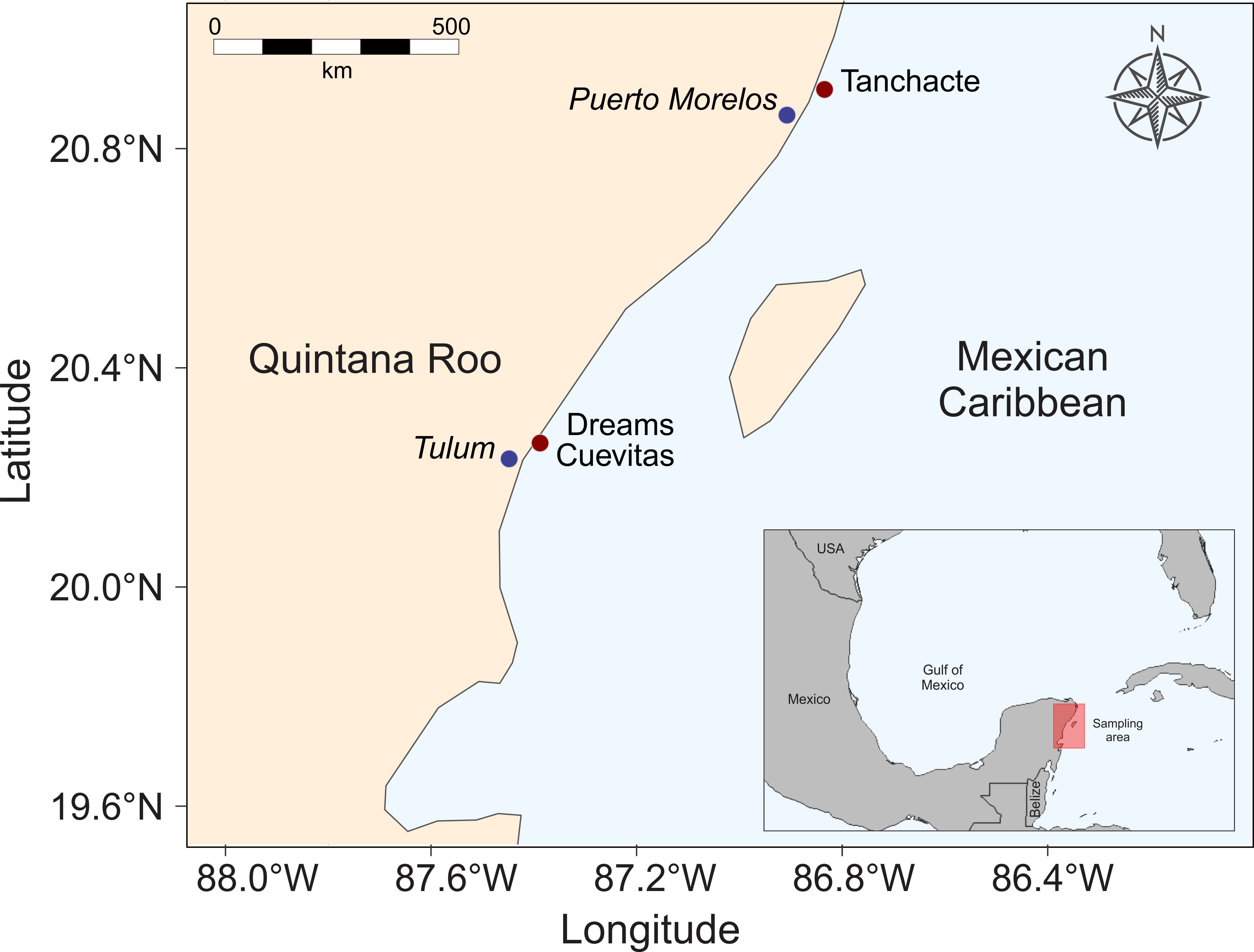

Supplement: S1 File — (ZIP) [file pone.0304925.s001.zip › S2_Fig1.jpg]

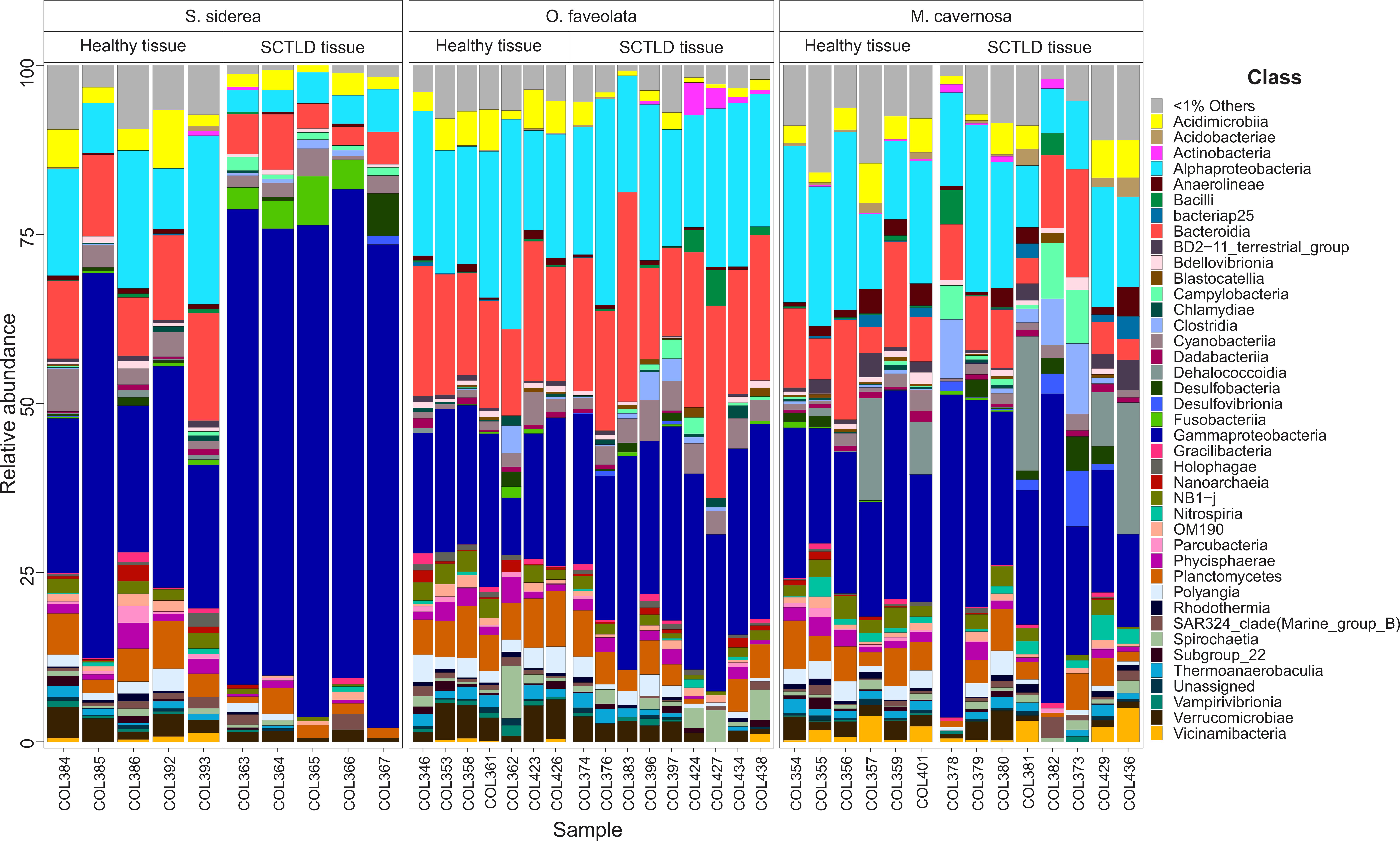

Supplement: S1 File — (ZIP) [file pone.0304925.s001.zip › S4_Fig2.jpg]

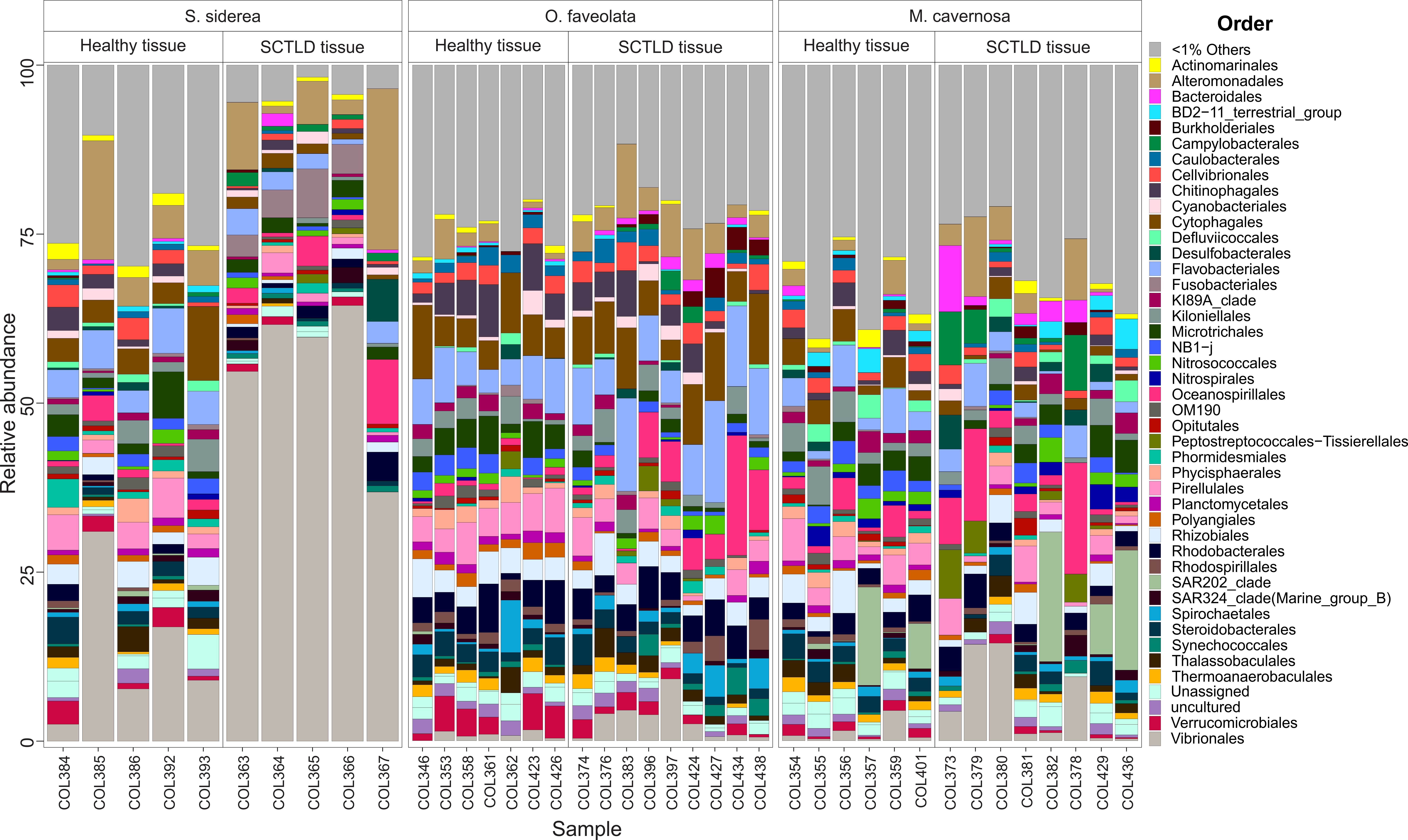

Supplement: S1 File — (ZIP) [file pone.0304925.s001.zip › S5_Fig3.jpg]

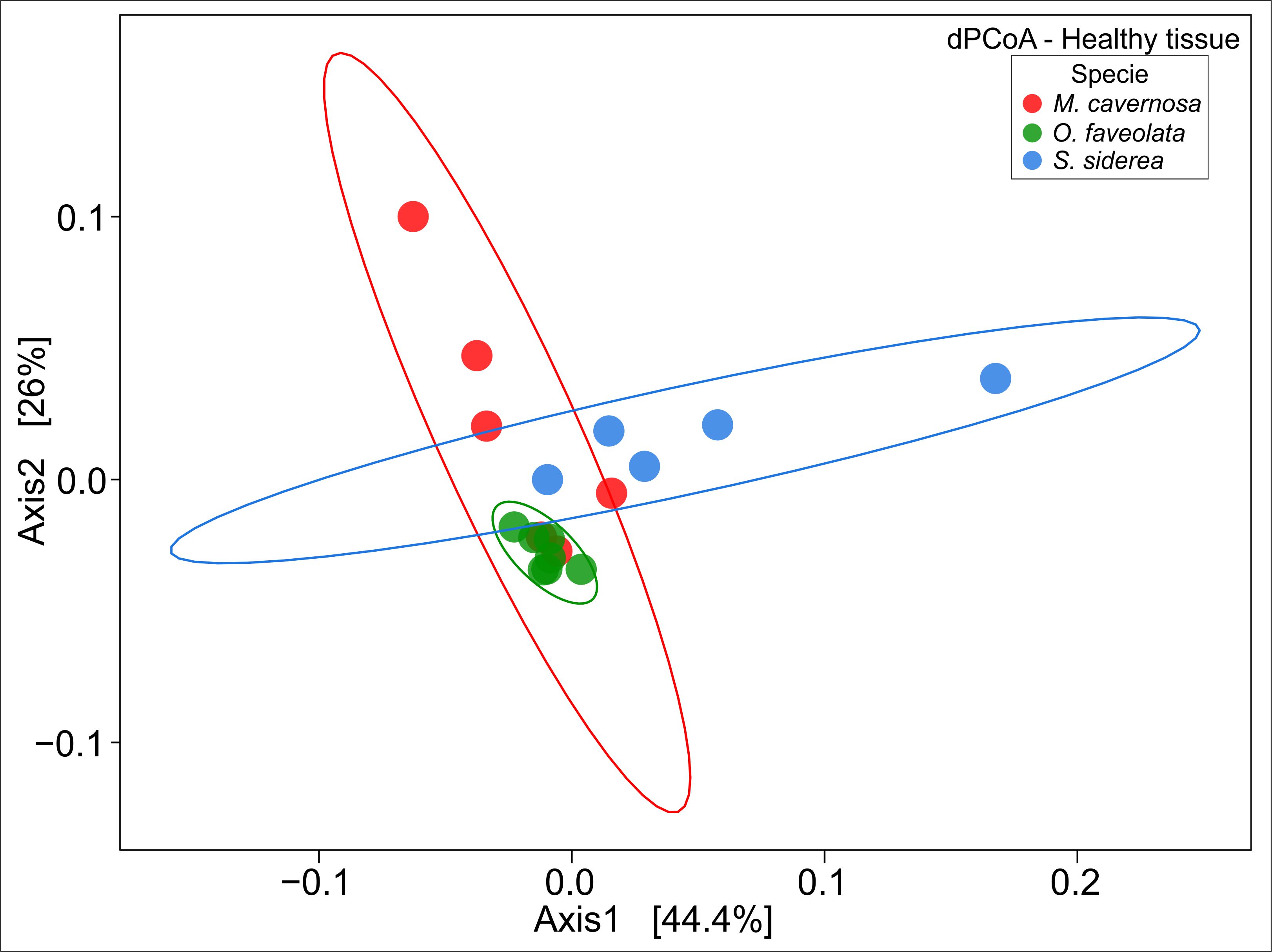

Supplement: S1 File — (ZIP) [file pone.0304925.s001.zip › S6_Fig4.jpg]

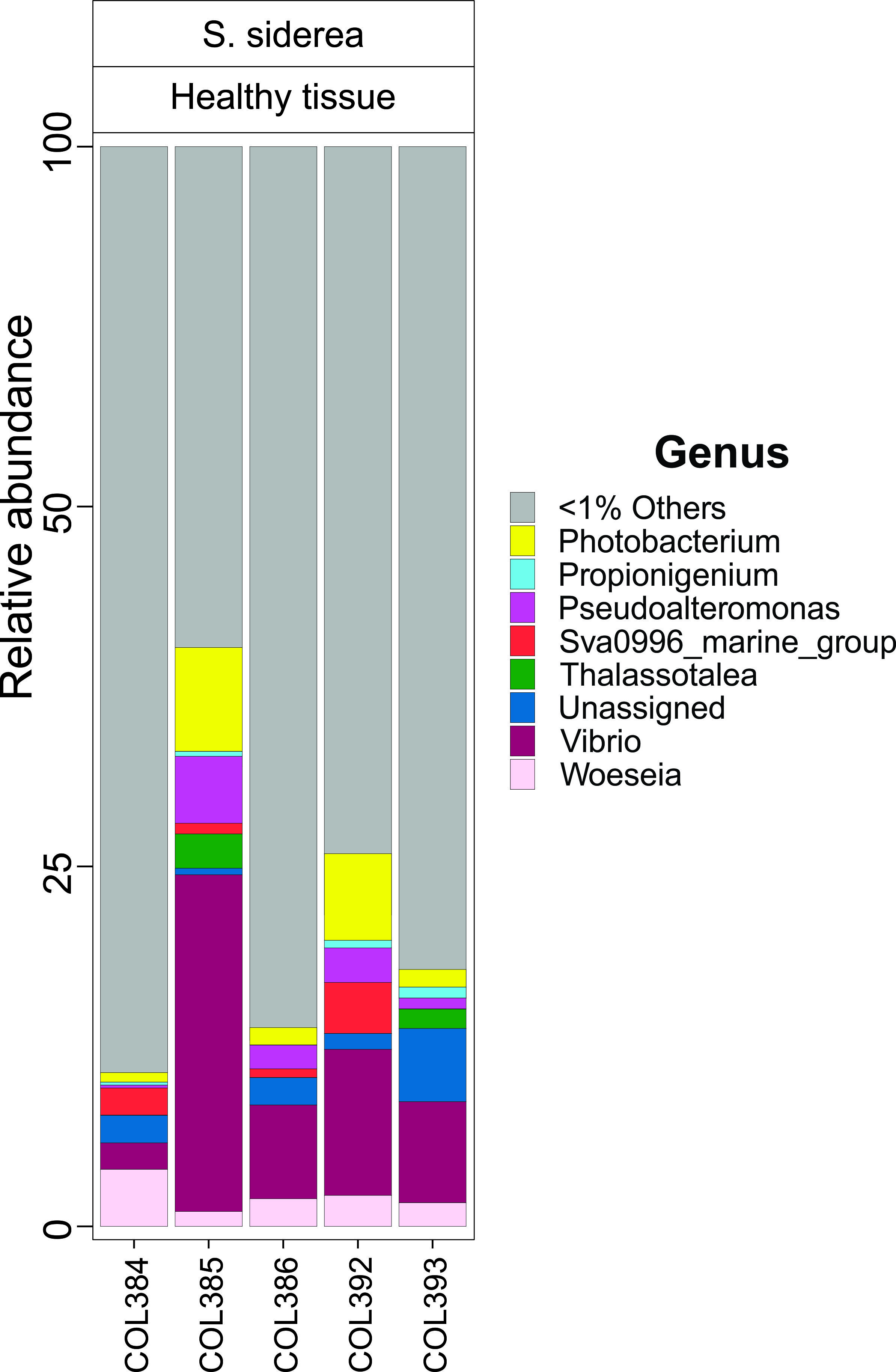

Supplement: S1 File — (ZIP) [file pone.0304925.s001.zip › S11_Fig5.jpg]
