# Supplementary material for: Microbiomes of three coral species in the Mexican Caribbean and their shifts associated with the Stony Coral Tissue Loss Disease
Source: PLoS One. 2024 Aug 26;19(8):e0304925. doi: 10.1371/journal.pone.0304925 (PMC11346732; doi:10.1371/journal.pone.0304925)
Supplement: Attachment — Submitted filename: MS_Zita_et_al_PONE_Responses.docx [file pone.0304925.s002.docx]

**Journal Requirements:**

Comment No 1: Please ensure that your manuscript meets PLOS ONE's style requirements.

**A**: We have made an additional revision of style requirements, and the manuscript meets the journal requirements.

Comment No 2: In your Methods section, please provide additional information regarding the permits you obtained for the work. Please ensure you have included the full name of the authority that approved the field site access and, if no permits were required, a brief statement explaining why.

**A:** Authority and permit were added.

“under collection permit issued to Universidad Nacional Autónoma de México (PPF/DGOPA-070/20).”

Comment No 3: Thank you for stating the following financial disclosure. Please state what role the funders took in the study. If the funders had no role, please state: "The funders had no role in study design, data collection and analysis, decision to publish, or preparation of the manuscript."

**A**: The funding institution, Consejo Nacional de Ciencia y Tecnología (CONACYT), had no role in study design, data collection and analysis, decision to publish, or preparation of the manuscript.

Comment No 4: Thank you for stating the following in the Acknowledgments Section of your manuscript. We note that you have provided funding information that is not currently declared in your Funding Statement. Please include your amended statements within your cover letter; we will change the online submission form on your behalf.

**A**: We eliminate the funding information from Acknowledgments Section. All the funding information was declared in the funding statement.

Comment No 5: We note that [S2_Fig 1] in your submission contain [map/satellite] images which may be copyrighted. We require you to either (1) present written permission from the copyright holder to publish these figures specifically under the CC BY 4.0 license.

**A**: We have provided information regarding the terms of use information for the map.

L616: “Data sources: (Administrative boundary layer: GADM database (www.gadm.org) under CC BY 4.0 license (https://gadm.org/license.html). The original maps were modified to show the study sites.”

Comment No 6: Please review your reference list to ensure that it is complete and correct.

**A**: A revision was made, and no retracted papers have been cited. The reference #18, in L480-482, was actualized.

**Review Comments to the Author**

Reviewer #1: The current study presents difference in diversity, composition and functional differences of microbial communities of 3 healthy and diseased coral species.

This paper is of sufficient scientific interest and originality in its contents. The authors have written it very well. Especially the discussion part is the strength of this manuscript.

**A**: Thank you for your comments.

Reviewer #2: It is not clear as to how amplicons from samples at various sites were pooled to produce sequencing libraries. How many samples contributed to each library?

**A**: In this study each library was prepared by sample and different samples from various sites weren’t pool to produce sequencing libraries. To avoid confusion, this information was modified. L126 and L136: “Library preparation per sample …” and “Sequencing libraries for the 16S rRNA gene were prepared according to the manufacturer’s protocol.”

What steps were performed to equalize sampling effort across libraries (rarefaction, etc.) Large read differences can result in incorrect diversity estimates and comparisons among samples. Please clarify.

**A**: We recognize that large differences can affect the diversity metrics. Thus, in this study the data set was normalized, as indicate L151-152: “The data set was normalized with the phyloseq library to obtain abundance tables…”

Minor: Typo in line 323- "can be result" should be "can result"

**A**: Done, this was corrected in L322.
